# Evolutionary conserved brainstem circuits encode category, concentration and mixtures of taste

**DOI:** 10.1038/srep17825

**Published:** 2015-12-07

**Authors:** Nuria Vendrell-Llopis, Emre Yaksi

**Affiliations:** 1 NERF, Leuven, Belgium; 2 KU Leuven, Leuven, Belgium; 3 VIB, Leuven, Belgium; 4 Kavli Institute for Systems Neuroscience and Centre for the Biology of Memory, Norwegian Brain Centre, Norwegian University of Science and Technology (NTNU), Trondheim, Norway

## Abstract

Evolutionary conserved brainstem circuits are the first relay for gustatory information in the vertebrate brain. While the brainstem circuits act as our life support system and they mediate vital taste related behaviors, the principles of gustatory computations in these circuits are poorly understood. By a combination of two-photon calcium imaging and quantitative animal behavior in juvenile zebrafish, we showed that taste categories are represented by dissimilar brainstem responses and generate different behaviors. We also showed that the concentration of sour and bitter tastes are encoded by different principles and with different levels of sensitivity. Moreover, we observed that the taste mixtures lead to synergistic and suppressive interactions. Our results suggest that these interactions in early brainstem circuits can result in non-linear computations, such as dynamic gain modulation and discrete representation of taste mixtures, which can be utilized for detecting food items at broad range of concentrations of tastes and rejecting inedible substances.

The sense of taste plays an indispensable role in the life of all animals. It is very important to monitor the chemical contents of ingested food in order to determine if it is palatable (and nutritive) or aversive (and toxic). For example, while the low level of sourness in a lemonade or slightly bitter taste of tonic water can be perceived as pleasant, strong sourness or bitterness can indicate the toxicity of food. In vertebrate brain, all these essential computations are performed by using only a few variables, namely the main categories of taste: sweet, salty, bitter, umami and sour.

Taste information from the gustatory receptor neurons are carried via taste fibers to the first relay station in the vertebrate brainstem. This evolutionary conserved brainstem regions are named facial lobe^1^ and vagal lobe^2^ in teleost fish, which are together related to the nucleus of the solitary tract in mammals^3^,^4^,^5^,^6^. These brainstem regions acts as a life support system and mediates vital taste related functions such as swallowing, salivation and appetitive behaviors^7^,^8^. Taste computations at the level of gustatory receptor neurons^9^,^10^,^11^,^12^,^13^,^14^,^15^ and the taste fibers^16^,^17^,^18^,^19^ are well studied. Moreover, thanks to several studies across different vertebrates, we know more and more about the processing of taste information in the vertebrate brainstem^20^,^21^,^22^,^23^,^24^,^25^,^26^,^27^,^28^,^29^,^30^ and gustatory cortex^31^,^32^. However, studying gustatory computations especially at the level of vertebrate brainstem has been extremely difficult in part due to the limited access of this brain structure for *in vivo* physiology in most vertebrate species.

In this study, we established a framework for studying the gustatory computations in the brainstem comprehensively by a combination of *in vivo* functional brain imaging, quantitative behavioral assays, and genetic tools in a small and optically transparent vertebrate, the zebrafish. Our results showed that taste categories are represented by dissimilar brainstem responses and generate different behaviors. We also showed that the concentrations of sour and bitter tastes are encoded differently, with different sensitivity and dose dependency. Moreover, we observed that mixing tastants leads to non-linear interactions, such as synergy and suppression. These interactions result in dynamic gain modulation, which increases the range of sensitivity for different taste concentrations. Finally, we observed that strong suppression of sour responses by bitter compounds leads to discrete taste mixture representations that resemble the stable states of neural attractors, which might serve to prevent ingestion of contaminated food items.

## Results and Discussion

**Figure 1:**
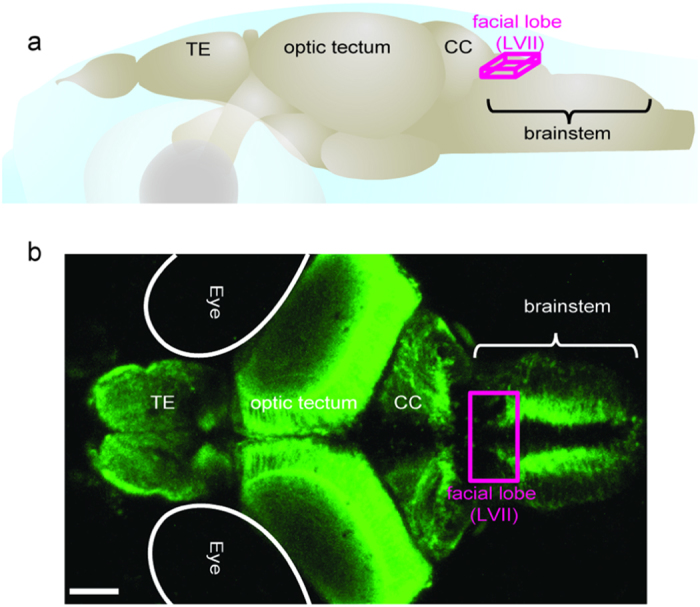
Schematic representation of zebrafish brain. (**a**) Illustration of 21 days old zebrafish brain. Magenta lines delineates the facial lobe of the brainstem, which is the main focus of this study. Major brain regions are labelled. Abbreviations: LVII facial lobe of the brainstem; CC, cerebellum; TE, telencephalon; (**b**) Maximum projection image of a 21dpf *elavl3*:GCaMP5 zebrafish brain across 320 μm depth. The magenta square indicates the facial lobe of zebrafish brainstem. Scale bar 100 μm.

**Figure 2:**
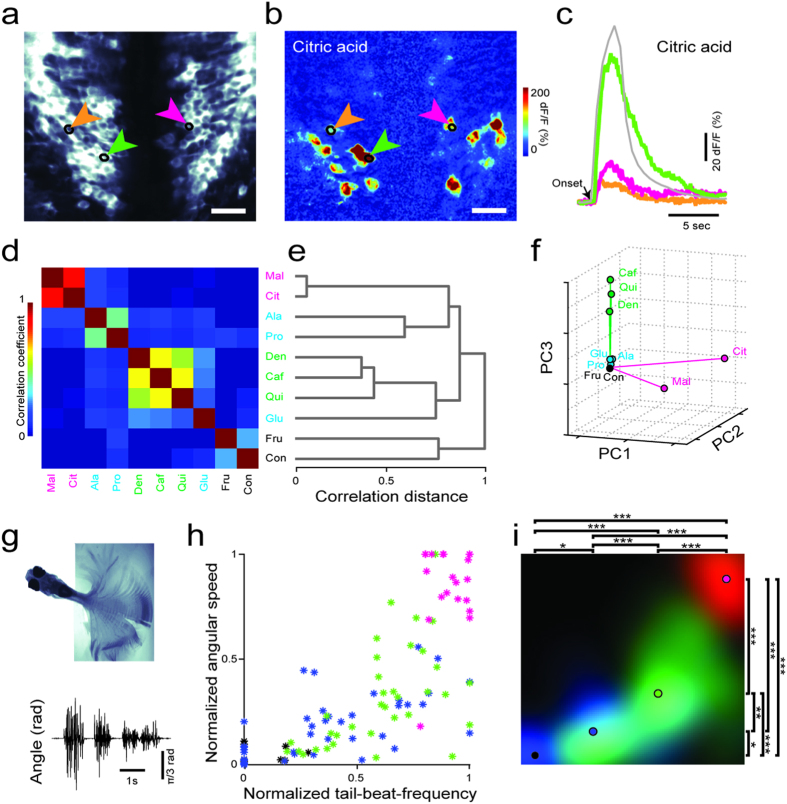
Taste categories are represented by dissimilar neural activity in the brainstem and generate different behaviors. (**a**) Optical section of the facial lobe in zebrafish brainstem expressing GCaMP5 under HuC promoter in most neurons. (**b**) Taste-evoked neural responses in the same optical section. Three arrows point to corresponding neurons from panel a. Scale bars 20 μm. Stimulus is citric acid 5 mM (**c**) Time course of the stimulus delivery in grey and time course of taste-evoked responses of example neurons color-coded same as in panel a. Black arrow indicates stimulus onset. (**d**) Pair-wise Pearson’s correlations between neural responses to different tastants. Text colors represent magenta = sour, green = bitter, blue = amino acids, black = sweet. n = 721 neurons, across 10 fishes. (**e**) Dendrogram showing the linkage among taste categories. Distances are measured based on correlations. (**f**) Representations of taste responses with the first 3 principle components. Total variance explained by first 3 principal components is 97.2% (**g**) Motor behavior of a semi-restrained zebrafish, obtained from a video sequence projected in time (top) and the associated tracking of the tail angle (bottom) in response to citric acid delivery. (**h**) TBF versus ATS in response to different taste categories, sour (magenta), bitter (green) and amino-acids (blue). Each dot represents an average of three different trials from individual fish (n = 12 fish) during the first four seconds. These values are normalized to the maximum response of each fish. Max ATS = 2.9 ± 1.2πrad/s, Max TBF = 24.2 ± 2.6 Hz. (**i**) Behavioral taste map obtained by filtering the graph in panel h. Colored circles represent averaged values across all fish. All categories generate significantly different behaviors. (***p = <0.0005, **p = <0.005, *p = <0.05 by Mann-Whitney U-test). For abbreviations, please see methods.

In order to study the gustatory computations in the zebrafish brainstem, we measured the neural responses evoked by taste stimuli in *elavl3:*GCaMP5 zebrafish expressing the transgenic calcium indicator GCaMP5 pan-neuronally^33^,^34^. We chose to perform all of our experiments in juvenile zebrafish (3–4 weeks old) that had been actively feeding for several weeks, yet still had transparent bodies. This selection had two major benefits: (1) the subjects had exposure to a variety of tastes while feeding prior to testing and (2) the permeability of light to their brain allowed two-photon calcium imaging to be performed without the need of surgery. In order to study the gustatory computations in the brainstem, we mainly focused our measurements on the zebrafish facial lobe (Figs 1 and 2a–c), which is homologous to the mammalian nucleus of the solitary tract^1^,^35^. We presented a broad set of tastants^11^,^36^ with different behavioral significances by using a computer controlled taste delivery system, while imaging the neural activity by a two photon microscope. The neuronal calcium signals in facial lobe neurons in response to the taste stimulation were robust and reproducible (Supplementary Figure 1,2). In order to investigate how taste categories are encoded, we presented a range of tastants from different taste categories and compared brainstem taste responses. For comparing multi-neuronal taste responses, we adopted several commonly used measures of similarity for neural activity, such as pairwise correlations (Fig. 2d, Supplementary Figure 3a), hierarchical clustering (Fig. 2e), principal component analysis (Fig. 2f) and Euclidean distances (Supplementary Figure 3b). All these similarity measures showed that tastants in the same category evoked similar neural activity and different taste categories are represented by dissimilar neural activity. To our surprise, while sour and bitter tastes evoked robust responses in the juvenile zebrafish facial lobe, amino acids that are typically strong taste stimuli for teleost fish^16^,^17^,^37^, evoked only weak responses. This can be due to the limited sensitivity of the calcium indicators or perhaps due to the developmental stage of the juvenile zebrafish that we examined, which was shown to be important for the assembly of the peripheral gustatory system in other teleosts^38^. In summary, our results revealed that the neural code for categorizing taste information at the level of the gustatory cortex^31^,^32^ is also present at the early gustatory centers of the brainstem. Hence, it is likely that these evolutionary conserved brainstem circuits can mediate several taste related behaviors before the taste information is passed to the next level in the brain.

Neural representations of gustatory information in the facial lobe suggest that zebrafish can distinguish different taste categories. We tested this hypothesis by measuring simple taste-evoked motor behaviors, such as angular tail speed (ATS) and tail beat frequency (TBF)^39^ in semi-restrained zebrafish^40^ (Fig. 2g). We choose to perform these behavioral experiments in semi-restrained animals in order to make sure that the stimulus conditions in these behavioral experiments match that of calcium imaging experiments. Here, we quantified that the zebrafish responded to amino acids with weak motor behaviors, whereas bitter tastants and sour tastants generated increasingly stronger motor behaviors, respectively (Fig. 2h,i). Consistent with the taste responses of facial lobe neurons in our experiments and behavioral studies in other fish species^41^,^42^,^43^ we observed that the zebrafish behave differently in response to different taste categories.

**Figure 3:**
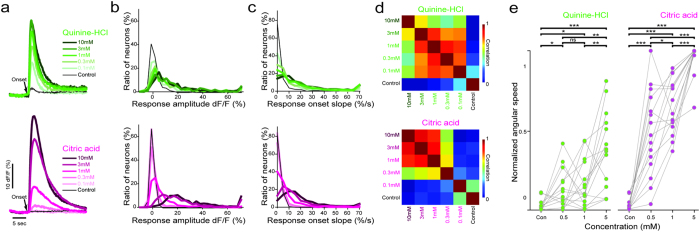
Taste concentrations are encoded differently for different taste categories. (**a**) Average response time course of facial lobe neurons in response to citric acid (magenta) and quinine-HCl (green) averaged across neurons. n = 830 neurons from 14 fish for citric acid and n = 329 neurons from 11 fish for quinine-HCl. Black arrow represents stimulus onset. Black lines are the responses of same neurons to blank control. Shades of colors represent different taste concentrations. (**b**) Histogram for the dose dependent response amplitude of individual neurons for the concentrations of quinine-HCl (above) and citric acid (below). Neural response amplitudes are averaged over the first 4 seconds after the stimulus onset. The response amplitude increase significantly for all concentrations of quinine-HCl and citric acid. (**c**) Histogram for the dose dependent response onset slopes of individual neurons for the concentrations of citric acid and quinine-HCl. Response onset slopes are calculated as the first coefficient of the first order polynomial, fitted to the rising phase of each neural response during the first 1.5 seconds. The response onset slope increase significantly for all concentrations of citric acid and quinine-HCl, except between quinine-HCl 3 mM and quinine-HCl 10 mM. (**d**) Pair-wise Pearson’s correlations between neural responses to different concentrations of quinine-HCl and citric acid. Note that the representations of low quinine-HCl concentrations are more similar to each other than low citric acid concentrations. (**e**) Motor behavior (normalized angular tail beat speed) of semi-restrained zebrafish in response to blank control and 3 different concentrations of quinine-HCl and citric acid. Max angular tail speed = 1.9 ± 0.7πrad/s. n = 15 fish (***p = <0.0005, **p = <0.005, *p = <0.05, ns = not significant. Mann-Whitney U-test for neural responses and Wilcoxon signed-rank test for motor behavior).

Stimulus intensity (concentration) can change the perceived taste quality. For example, humans, rodents and teleost fish perceive low concentrations of sour taste as a feeding cue^36^,^44^,^45^,^46^, and high concentrations of sour taste as unpleasant^44^,^45^. Moreover, different taste categories have been shown to have different detection thresholds^47^; for example, bitter compounds can be detected at lower concentrations^48^,^49^. To test how taste intensity is encoded in the brainstem, we measured the responses of facial lobe neurons to a range of concentrations within the two taste categories that we observed strong neural and behavioral responses, the sour and the bitter taste. Here, we specifically chose taste concentration ranges that were used in previous studies performed in teleost fish^11^ and in rodents^50^. Our results showed that the bitter tastants induce strong responses in the bitter-sensitive facial lobe neurons, even at low concentrations, and there is only a small but significant increase in the response amplitude or the ratio of responding neurons with increasing bitter concentration (Fig. 3a,b). On the other hand, sour tastants did not elicit strong responses at low concentrations, but increasing the sour taste concentrations resulted in significantly larger responses and recruitment of more responding neurons (Fig. 3a,b). Interestingly, we also observed a significant increase in the response onset slope for the increasing concentrations of both sour and bitter responses (Fig. 3c). These results suggest that both the amplitude and the onset slope of the taste responses in facial lobe neurons can carry information about taste concentration to the downstream brain regions. We should also note that while most neurons follow these general rules for encoding taste concentrations, we also observed small numbers of neurons with complex dose-dependent responses (Supplementary Figure 4), which suggest strong nonlinear interactions in the facial lobe circuits. It is likely that the gustatory circuits make use of these diverse types of neurons with different taste sensitivities in order to cover a larger dynamic range for encoding taste concentration.

The amplitude and the onset of neural responses to varying sour and bitter taste concentrations suggest that the stimulus intensity for these different taste categories might be encoded by different principles. In order to test this, we compared the multi-neuronal responses to sour and bitter taste concentrations by using pairwise correlations of neural activity. We observed that sour concentrations are encoded by more dissimilar neural activity than bitter taste, especially at low concentrations (Fig. 3d). These differences in the neural encoding of sour and bitter taste concentrations suggest that the zebrafish can distinguish between sour taste concentrations more readily than bitter taste concentrations. We tested this hypothesis by measuring the motor behavior of semi-restrained zebrafish in response to various sour and bitter taste concentrations. Consistent with the neuronal responses, we observed that the animals can behaviorally differentiate concentrations of sour taste better than the concentrations of bitter taste (Fig. 3e). Overall, our results suggest that concentrations of sour and bitter tastes are encoded differently in the facial lobe of the brainstem, which results in a better separation for the sour taste concentrations when compared to bitter taste concentrations. One hypothesis is that it is more relevant for the taste circuits to be sensitive to low levels of bitterness, which signal toxins, rather than encoding precise bitter concentrations. Alternatively, it is likely that different levels of sourness might have different qualities ranging from edible tasty food to inedible irritants, which is a very important distinction to make.

**Figure 4:**
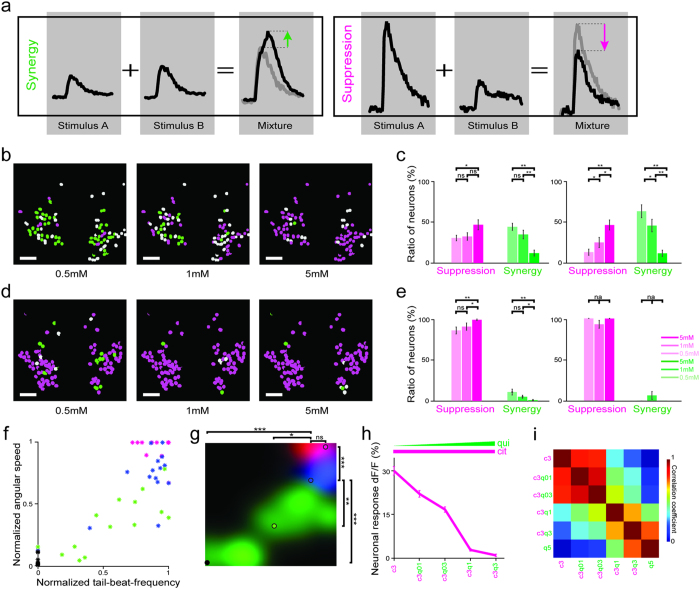
Taste mixtures exhibit suppressive and synergistic interactions, which are dynamically modulated by the taste concentration and category. (**a**) Schematic representation for quantifying mixture interactions. T**a**ste responses of two representative neurons, which show synergy (left) and suppression (right) respectively. Individual and mixtures taste responses(black) and predictions based on synergy and suppression rules as described in methods (grey). (**b**) Representative facial lobe neurons from individual zebrafish are color coded by the mixture interactions they exhibit (suppression = magenta, synergy = green, rest = grey) in response to mixtures of sour tastants (citric acid and malic acid) at different concentrations. (**c**) Ratio of all neurons (left) and responding neurons (right) exhibiting synergy or suppression changes dynamically with changing concentrations. N = 1129 neurons in 7 fish. (**d**) Representative facial lobe neurons are color coded for suppressive and synergistic interactions for the mixtures of sour (citric acid) and bitter (quinine-HCl) taste. White scale bars are 20 μm for b and d. (**e**) Ratio of all neurons (left) and responding neurons (right) exhibiting synergy or suppression for sour/bitter mixtures (for c and e ***p = <0.0005, **p = <0.005, *p < 0.05 by Wilcoxon signed-rank test, ns = not significant, n.a = not applicable). N = 490 neurons in 7 fish. Error bars are standard error of the mean across fish for c and e. (**f**) TBF and ATS of zebrafish in response to citric acid (magenta), quinine-HCl (green) and their mixture (blue) at 5 mM concentrations. Each dot represents a three trial average from individual fish (n = 15 fish) during first four seconds. Max ATS = 1.9 ± 0.7πrad/s. Max TBF = 24.8 ± 4.0 Hz. (**g**) Behavioral taste map obtained by Gaussian filtering the graph in f. Colored circles represent average values across all fish. All combinations generate significantly different behaviors (***p = <0.0005, **p = <0.005, *p < 0.05 by Wilcoxon signed-rank test, ns = not significant). (**h**) Dose dependent average amplitude for the neural responses to mixtures of citric acid and quinine-HCl. Citric acid concentration is kept at 3 mM and quinine-HCl concentration is gradually increased morphing the mixture responses. Error bars are standard error of the mean. (**i**) Pair-wise Pearson’s correlations between neural responses to morphing mixtures of citric acid and quinine-HCl. Note the abrupt change in the pairwise similarity of neural responses. N = 259 neurons in 10 fish.

Our food is usually a mixture of different tastes and the presence of one taste category can alter the perceived quality of the other tastants within the mixture^51^,^52^. Several recent studies suggest that these kind of mixture interactions across taste categories are absent at the early levels of mammalian gustatory pathway, such as in gustatory receptor neurons and the taste fibers^53^,^54^,^55^. In teleosts fish, however, several types of taste mixture interactions were shown to be present^56^,^57^,^58^,^59^ already at the level of taste receptors and taste fibers. It is unclear whether such interactions are also prominent at the next level of the gustatory pathway, the facial lobe of the zebrafish brainstem. To test potential taste mixture interactions across facial lobe neurons responding to different taste categories, we delivered mixtures of tastes and analyzed interactions of these taste responses with each other using simple and previously used measures of synergy and suppression (Fig. 4a). In brief, if taste mixture responses were larger than the linear summation of the neuronal responses to individual tastes, we labelled these as synergy interactions^60^. If taste mixture responses were smaller than the biggest of the individual components, we labeled these as suppression interactions^60^. Using this simple analysis on the neural data we observed both suppressive and synergistic interactions for the mixtures of sour tastes. Our analysis revealed that the probability of observing synergistic or suppressive interactions across sour tastes is highly dependent on the taste concentration. We observed that sour mixtures at low concentrations predominantly led to synergy, whereas sour mixtures at high concentrations mostly resulted in suppression (Fig. 4b,c). It is possible that the concentration dependency for the observed synergy among sour taste mixtures can in part be due to potential saturation in the dose response curves of individual neurons. However, our observation for the dynamic modulation of suppression is independent of these dose-dependent effects. In either case, our results suggested that the gain of brainstem circuits for encoding taste information can be dynamically modulated by tuning these synergistic and suppressive interactions. At low concentrations, sour taste mixtures interact synergistically and this increases the sensitivity of the gustatory system, which can help detect low sour concentrations, which are shown to be important as feeding cues that help to locate prey or food in other teleosts^36^,^46^. In contrast, when the concentration is high, we predominantly observe suppression in sour taste mixtures, which likely prevents the saturation of taste responses by reducing the sensitivity of the gustatory system. Consistent with our results in the brainstem, such gain modulation for taste responses was shown to persist at the latter stage of the taste pathway at the level of the insular cortex^61^ of mammals. It will be interesting to test the impact of these non-linear taste computations also in the teleost equivalent of insular cortex located in the forebrain. We propose that the dynamic gain modulation we observed at the level of brainstem neurons can extend the range of gustatory coding across a wider range of stimulus intensities, from very low to very high taste concentrations. Hence, this dynamic modulation of gain would allow the taste circuits to adjust their sensitivity according to the needs of the changing chemical environment where a low level of sour taste can act as a feeding cue^36^,^46^ and high sourness might signal a potentially acidic and thus hazardous location.

Bitter tastes, which are often associated with toxins, may suppress the neural responses to other taste categories in the peripheral gustatory system of rodents^62^ and insects^63^. It is unclear whether such a suppressive effect of bitter tastes is also prominent at the next levels of gustatory pathway. To study this phenomenon, we measured the mixture interactions between bitter and sour tastants in the zebrafish facial lobe. We observed that the bitter tastants strongly suppressed the neural responses to sour taste (Supplementary Figure 5). We observed that while all bitter tastants used in this study (denatonium, quinine-HCl and caffeine) suppressed sour taste responses, quinine-HCl showed the strongest effect (Supplementary Figure 5a); thus, we continued our further investigations focusing on the mixtures of bitter taste quinine-HCl and sour tastants. Unlike the mixture interactions between sour tastants which are concentration dependent, the suppression of sour taste by the bitter taste was prominent at all concentrations (Fig. 4d,e). In line with this suppression, we also confirmed that motor response of juvenile zebrafish to the sour/bitter mixture is weaker than the motor response to sour taste alone (Fig. 4f,g). These results showed that a few number of bitter sensitive neurons with robust responses are sufficient to suppress a large portion of sour sensitive neuron, which highlight the importance of this mechanism that ensures to keep zebrafish away from a potential source of toxic bitter substances. It is likely that such strong suppression by bitter compounds on the sour taste responses might be related to a highly conserved neural mechanism, which evolved to prevent the ingestion of toxic substances that could contaminate the food items.

Several sensory modalities, such as olfaction^64^, audition^65^ and vision^66^, were shown to be encoded in a discrete manner by abruptly changing from one neural representation to another, resembling the stable states of neural attractors. These discrete transitions between stables states are suggested to be important for efficient stimulus discrimination in highly noisy environments and degraded stimulus conditions. Yet, it is unclear whether such discrete encoding of taste can occur in the gustatory system. To test whether discrete transitions also exist for the encoding of taste stimuli, we morphed the composition of sour/bitter mixtures by gradually increasing the concentration of the bitter compound (Fig. 4h). We observed that with low bitter concentrations, the sour/bitter mixture responses are highly correlated to the neural responses to sour compounds (Fig. 4i), despite the prominent inhibition of sour responses by bitter tastants (Fig. 4h). However, the similarity of the sour/bitter mixture to sour compounds decreased abruptly by increasing the bitter concentration, which made the sour/bitter mixture responses more similar to bitter compounds (Fig. 4i, Supplementary Figure 5b,c). Our results suggest that the discrete neural representations that were shown in other sensory systems are also present in the gustatory system. It is likely that these discrete sensory representations are universal computations, which might be utilized to serve different functions for different sensory modalities. In the case of taste circuits, this sudden shift of neural responses for different sour/bitter taste mixture combinations may represent two distinct states in the facial lobe networks, edible and inedible. Our results suggest that the low levels of bitterness in taste mixtures do not significantly change the responses to sour taste and, therefore, are tolerable. However medium or high bitter concentrations in the sour/bitter mixtures, which might correspond to toxicity, can suddenly shift the neural representations of sour/bitter mixtures to be similar to those of bitter compounds and prevent ingestion of inedible food. It was shown that the gustatory system is plastic and can change as the animals age^38^. It is likely that these distinct shifts in taste representations that we observed in juvenile zebrafish are highly species and age specific.

In summary, our results revealed that the brainstem circuits can encode taste category and concentration, and mediate the interactions between mixtures of tastants. We propose that these evolutionary conserved circuits can utilize universal neural mechanisms, such as dynamic gain modulation and discrete neural representations, in order to perform complex gustatory computations and execute gustatory behaviors, before the taste information is passed to the higher brain regions. It will now be interesting to study the local circuit architecture within the facial lobe of the brainstem that could underlie some of these computations and to understand how these sensory circuits interact with the motor circuits, which are controlling animal behavior.

## Experimental Procedures

### Zebrafish

The experiments were performed on size-matched juvenile zebrafish (*Danio Rerio*) at 18–24 dpf, expressing GCamp5^67^ under *elavl3(*HuC)^68^ promoter. No surgery was required since the zebrafish were in transparent nacre (*mitfa* mutant) background. Fish were kept at a density of 10–15 per 3.5 liter tanks and maintained under a 14/10 day/night cycle in a closed circulating system (Technilab-BMI bv) at 28.5 Celcius. *Preparation.* For calcium imaging experiments, the fish were first anaesthetized with 0.02% MS222, then embedded in 1% low melting point agarose (LMP) in a recording chamber (Fluorodish, Wold Precision Instruments), and subsequently paralyzed using a alphabungaro-toxin injection (Invitrogen). Later, the LMP obstructing the mouth was removed. For the behavioral experiments, we first embedded the fish in LMP in a small Fluorodish chamber, and then removed the LMP obstructing the mouth and the tail, allowing free tail movements. The setup was constantly perfused with fresh, oxygenated artificial fish water (AFW: 1.2 g of sea salt in 20 L reverse osmosis (RO) water) throughout all the experiments. All animal procedures were performed in accordance with the animal care guidelines issued by the government of Belgium. Please see the ethical statement below for the license number.

### Tastants

The tastants were dissolved in AFW. Solutions were stored at 4 °C for not more than one week. 50 ml stocks of 200 mM of amino acids and 50 ml stocks of 20 mM of bitter and sour compounds were kept at −20 degrees. All tastants were provided by Sigma-Aldrich (Belgium).

For the taste category experiments, we used a panel of nine tastants, as well as a control (AFW), which included: Mal: malic acid 10 mM; Cit: citric acid 10 mM; Ala: L-Alanine 100 mM; Pro: L-Proline 100 mM; Den: denatonium benzoate, 10 mM; Caf: caffeine 10 mM; Qui: quinine-HCl 10 mM; Glu: glutamic acid 100 mM; Fru: fructose 100 mM. For the taste concentrations experiment we used 0.1, 0.3, 1, 3 and 10 mM of citric acid (sour) and quinine-HCl (bitter) for the calcium imaging experiments, and the same tastants in 0.5, 1 and 5 mM concentrations for the behavioral experiments. In the binary mixture experiments of citric and malic acid, as well as citric acid and quinine-HCl, we used 0.5, 1 and 5 mM of each tastant, including their final concentration in the mixture solution (Fig. 4a–g). For morphing the citric acid and quinine-HCl mixture (Fig. 4h–i) we gradually increased the concentration of quinine-HCl in the mixtures (0–0.1 mM-0.3 mM-1 mM-3 mM) while citric acid concentration was kept constant at 3 mM. Supplementary Table 1 reports the pHs for all used tastants.

Throughout all of the experiments we used a constant stream (100 ml/h) of AFW directed to the mouth. Tastants were added into this stream with the switching of a computer-controlled 6 port 2-position valve (Valco Instruments). The time course of the taste stimulus was monitored by application of fluorescein solution. Each tastant was delivered three times in a randomized order and successive applications were separated by 2 min to avoid adaptation.

### Functional brain imaging

Images were acquired using a two-photon microscope and 20× water immersion objective (Zeiss). A femtosecond Ti:Sapphire laser (Spectra Physics) was used as an excitation source at 920 nm. Images were acquired using Zeiss software at 7.5 Hz from a single optical plane at a time. We collected data from up to three different optical planes (with 10 μm separation) of the facial lobe in each juvenile zebrafish, covering a large portion of the facial lobe. For ensuring the reliability and robustness of our conclusions we used large sample size of juvenile zebrafish in all our imaging experiments. Here we used n = 10 fish for taste categories, n = 14 fish and n = 11 fish for taste concentrations, n = 7 fish for taste mixtures and n = 10 fish for taste mixture morphing experiments.

### Behavioral experiments

Video during behavioral experiments was acquired using a fast camera (Exg03 Baumer) at 100 Hz and the MATLAB Imaging acquisition toolbox. The larvae were acclimated for at least 1 hour in the setup prior to data collection. All experiments were conducted during the afternoon at ambient room temperature (~25°). For these behavioral experiments we used n = 12 fish for taste categories and n = 15 fish for taste mixture experiments.

### Data analysis

Two-photon fluorescence images were first aligned using an algorithm that corrects occasional XY drift. Individual neurons were identified by a pattern recognition algorithm, which automatically finds neurons by using a correlation based approach for comparing the Gcamp5 labelled neurons with torus shaped neuronal templates of iterated sizes^69^. Later these automatically detected neurons are confirmed by manual inspection of a human user. Next, identified and confirmed neurons on the images are segmented into regions of interest (ROIs) as described before^33^. The relative change in fluorescence (dF/F) is calculated in pixels corresponding to individual neurons (ROIs). The results from three randomized trials for each stimulus were averaged. We only analyzed the neurons responding to at least one of the presented taste stimulus larger than 4 standard deviations or smaller than 3 standard deviations from the baseline period, which is one second preceding the stimulus delivery.

To analyze the behavior responses of the zebrafish, we first tracked the tail and then calculated the angular tail speed (ATS) and tail beat frequency (TBF). The ATS corresponds to the average angular displacement of the tail over 4 seconds after stimulus delivery. The TBF was calculated as the inverse of the time required for the tail to bend in a complete cycle (left-right)^39^. The ATS and TBF metrics were averaged across three trials, and normalized by the maximum ATS and TBF of each individual fish. Mann-Whitney U-test was used to study the significance of the results while comparing categories of tastants due to diverse sample size, whereas Wilcoxon paired test was used while comparing mixture interactions.

Please see the Supplementary table 2 for the detailed description of the sample size and the significance values for comparative tests

### Multi-variate statistical tools

To study how diverse or similar the neuronal responses evoked by different tastants were, meaning the linear dependence for the patterns of neural activity across different tastants, we calculated the Pearson correlation coefficients shown as correlation matrices in the figures. We also performed PCA, which uses an orthogonal transformation to identify the most relevant axis with highest variance. Then, we converted the first three principal components into coordinates to visualize a clear representation of variance across tastants. To study the difference on temporal reaction based on the onset slope of neuronal responses, we fit a first-order polygon curve to the first 1.5 seconds of the evoked response and used the first coefficient to quantify and compare the different slopes.

### Mixture interaction analysis

To analyze the mixture interactions we defined synergy as any response to a binary mixture that is bigger than the summation of the individual components: R(a + b) > R(a) + R(b)^60^,^62^.

Suppression was defined as any response to a binary mixture that is smaller than the biggest individual response: R(a + b) < max(R(a),R(b))^60^,^61^.

To study the significance of the results we used a non-parametric statistical test for repeated measurements on a single sample of neurons, known as Wilcoxon signed-rank test.

### Ethical Statement for animal procedures and experimental protocols

All animal procedures and all experimental protocols in this manuscript were approved by the Ethical Committee of KULeuven by the license number P133/2013, in accordance with the animal care guidelines issued by the government of Belgium.

## Additional Information

**How to cite this article**: Vendrell-Llopis, N. and Yaksi, E. Evolutionary conserved brainstem circuits encode category, concentration and mixtures of taste. *Sci. Rep.* **5**, 17825; doi: 10.1038/srep17825 (2015).

## Supplementary Material

Supplementary Information

## References

[b1] Finger T. E. Gustatory pathways in the bullhead catfish. 1. Connections of the anterior ganglion. J Comp Neurol 165, 513–526 (1976). 1262542 10.1002/cne.901650407

[b2] Huesa G., Ikenaga T., Bottger B. & Finger T. E. Calcium-fluxing glutamate receptors associated with primary gustatory afferent terminals in goldfish (Carassius auratus). J Comp Neurol 506, 694–707 (2008). 18067143 10.1002/cne.21571 PMC2543131

[b3] Di Lorenzo P. M. The neural code for taste in the brain stem: response profiles. Physiol Behav 69, 87–96 (2000). 10854920 10.1016/s0031-9384(00)00191-8

[b4] Travers J. B., Travers S. P. & Norgren R. Gustatory neural processing in the hindbrain. Annu Rev Neurosci 10, 595–632 (1987). 3551765 10.1146/annurev.ne.10.030187.003115

[b5] Norgren R., Nishijo H. & Travers S. P. Taste responses from the entire gustatory apparatus. Ann N Y Acad Sci 575, 246–263; discussion 263–244 (1989). 2699190 10.1111/j.1749-6632.1989.tb53248.x

[b6] Purves D. *et al.* Neuroscience (Sinauer Associates, Sunderland, Mass, 2012).

[b7] Grill H. J. & Norgren R. The taste reactivity test. II. Mimetic responses to gustatory stimuli in chronic thalamic and chronic decerebrate rats. Brain Res 143, 281–297 (1978). 630410 10.1016/0006-8993(78)90569-3

[b8] Finger T. E. Evolution of gustatory reflex systems in the brainstems of fishes. Integr Zool 4, 53–63 (2009). 20160963 10.1111/j.1749-4877.2008.00135.x PMC2759750

[b9] Chandrashekar J., Hoon M. A., Ryba N. J. & Zuker C. S. The receptors and cells for mammalian taste. Nature 444, 288–294 (2006). 17108952 10.1038/nature05401

[b10] Kapsimali M. & Barlow L. A. Developing a sense of taste. in Seminars in cell & developmental biology Vol. 24, 200–209 (Elsevier, 2012). 23182899 10.1016/j.semcdb.2012.11.002 PMC3604069

[b11] Oike H. *et al.* Characterization of ligands for fish taste receptors. J Neurosci 27, 5584–5592 (2007). 17522303 10.1523/JNEUROSCI.0651-07.2007 PMC6672760

[b12] Ohmoto M., Okada S., Nakamura S., Abe K. & Matsumoto I. Mutually exclusive expression of GÎ ± ia and GÎ ± 14 reveals diversification of taste receptor cells in zebrafish. Journal of Comparative Neurology 519, 1616–1629 (2011). 21452212 10.1002/cne.22589 PMC3394409

[b13] Aihara Y. *et al.* Transgenic labeling of taste receptor cells in model fish under the control of the 5′-upstream region of medaka phospholipase C-beta 2 gene. Gene Expression Patterns 7, 149–157 (2007). 16920036 10.1016/j.modgep.2006.06.004

[b14] Matsumoto I., Ohmoto M. & Abe K. Functional diversification of taste cells in vertebrates. in Seminars in cell & developmental biology Vol. 24, 210–214 (Elsevier, 2013). 23085625 10.1016/j.semcdb.2012.10.004 PMC3594521

[b15] Herrick C. J. The organ and sense of taste in fishes Vol 22, 237–272 (Bull. U.S. Fish. Comm, 1903).

[b16] Caprio J. Olfaction and taste in the channel catfish: an electrophysiological study of the responses to amino acids and derivatives. Journal of comparative physiology 123, 357–371 (1978).

[b17] Caprio J. Electrophysiological distinctions between the taste and smell of amino acids in catfish. Nature 266, 850–851 (1977). 865608 10.1038/266850a0

[b18] Kanwal J., Hidaka I. & Caprio J. Taste responses to amino acids from facial nerve branches innervating oral and extra-oral taste buds in the channel catfish, Ictalurus punctatus. Brain research 406, 105–112 (1987). 3567621 10.1016/0006-8993(87)90774-8

[b19] Marui T. & Caprio J. Teleost gustation. in Fish chemoreception 171–198 (Springer, 1992).

[b20] Di Lorenzo P. M. & Victor J. D. Taste response variability and temporal coding in the nucleus of the solitary tract of the rat. Journal of Neurophysiology 90, 1418–1431 (2003). 12966173 10.1152/jn.00177.2003

[b21] Lemon C. H. & Smith D. V. Neural representation of bitter taste in the nucleus of the solitary tract. Journal of Neurophysiology 94, 3719–3729 (2005). 16107527 10.1152/jn.00700.2005

[b22] Di Lorenzo P. M. & Victor J. D. Neural coding mechanisms for flow rate in taste-responsive cells in the nucleus of the solitary tract of the rat. Journal of Neurophysiology 97, 1857–1861 (2007). 17182909 10.1152/jn.00910.2006 PMC2659613

[b23] Roussin A. T., D'Agostino A. E., Fooden A. M., Victor J. D. & Di Lorenzo P. M. Taste coding in the nucleus of the solitary tract of the awake, freely licking rat. The Journal of neuroscience 32, 10494–10506 (2012). 22855799 10.1523/JNEUROSCI.1856-12.2012 PMC3427930

[b24] Travers S. P. & Geran L. C. Bitter-responsive brainstem neurons: characteristics and functions. Physiology & behavior 97, 592–603 (2009). 19303890 10.1016/j.physbeh.2009.02.042

[b25] Kinzeler N. R. & Travers S. P. u-Opioid modulation in the rostral solitary nucleus and reticular formation alters taste reactivity: evidence for a suppressive effect on consummatory behavior. American Journal of Physiology-Regulatory, Integrative and Comparative Physiology 301, R690–R700 (2011). 10.1152/ajpregu.00142.2011 PMC3174751 21697523

[b26] Ikenaga T., Ogura T. & Finger T. E. Vagal gustatory reflex circuits for intraoral food sorting behavior in the goldfish: cellular organization and neurotransmitters. J Comp Neurol 516, 213–225 (2009). 19598285 10.1002/cne.22097 PMC2737690

[b27] Sharp A. A. & Finger T. E. GABAergic modulation of primary gustatory afferent synaptic efficacy. Journal of neurobiology 52, 133–143 (2002). 12124751 10.1002/neu.10073

[b28] Smeraski C. A., Dunwiddie T. V., Diao L. & Finger T. E. Excitatory amino acid neurotransmission in the primary gustatory nucleus of the goldfish Carassius auratus. Annals of the New York Academy of Sciences 855, 442–449 (1998). 10049227 10.1111/j.1749-6632.1998.tb10604.x

[b29] Kotrschal K. & Finger T. E. Secondary connections of the dorsal and ventral facial lobes in a teleost fish, the rockling (Ciliata mustela). The Journal of comparative neurology 370, 415–426 (1996). 8807445 10.1002/(SICI)1096-9861(19960708)370:4<415::AID-CNE1>3.0.CO;2-7

[b30] Goehler L. E. & Finger T. E. Functional organization of vagal reflex systems in the brain stem of the goldfish, Carassius auratus. Journal of Comparative Neurology 319, 463–478 (1992). 1619041 10.1002/cne.903190402

[b31] Chen X., Gabitto M., Peng Y., Ryba N. J. & Zuker C. S. A gustotopic map of taste qualities in the mammalian brain. Science 333, 1262–1266 (2011). 21885776 10.1126/science.1204076 PMC3523322

[b32] Accolla R., Bathellier B., Petersen C. C. H. & Carleton A. Differential spatial representation of taste modalities in the rat gustatory cortex. The Journal of neuroscience 27, 1396–1404 (2007). 17287514 10.1523/JNEUROSCI.5188-06.2007 PMC6673570

[b33] Jetti S. K., Vendrell-Llopis N. & Yaksi E. Spontaneous activity governs olfactory representations in spatially organized habenular microcircuits. Curr Biol 24, 434–439 (2014). 24508164 10.1016/j.cub.2014.01.015

[b34] Ahrens M. B., Orger M. B., Robson D. N., Li J. M. & Keller P. J. Whole-brain functional imaging at cellular resolution using light-sheet microscopy. Nature methods 10, 413–420 (2013). 23524393 10.1038/nmeth.2434

[b35] Butler A. B. & Hodos W. Comparative vertebrate neuroanatomy: evolution and adaptation (John Wiley & Sons, 2005).

[b36] Goli S., Jafari V., Ghorbani R. & Kasumyan A. Taste preferences and taste thresholds to classical taste substances in the carnivorous fish, kutum Rutilus frisii kutum (Teleostei: Cyprinidae). Physiol Behav 140, 111–117 (2015). 25497081 10.1016/j.physbeh.2014.12.022

[b37] Valentincic T., Lamb C. F. & Caprio J. Expression of a reflex biting/snapping response to amino acids prior to first exogenous feeding in salmonid alevins. Physiol Behav 67, 567–572 (1999). 10549895 10.1016/s0031-9384(99)00112-2

[b38] Finger T. E., Drake S. K., Kotrschal K., Womble M. & Dockstader K. C. Postlarval growth of the peripheral gustatory system in the channel catfish, Ictalurus punctatus. J Comp Neurol 314, 55–66 (1991). 1797874 10.1002/cne.903140106

[b39] Budick S. A. & O'Malley D. M. Locomotor repertoire of the larval zebrafish: swimming, turning and prey capture. J Exp Biol 203, 2565–2579 (2000). 10934000 10.1242/jeb.203.17.2565

[b40] Wyart C. *et al.* Optogenetic dissection of a behavioural module in the vertebrate spinal cord. Nature 461, 407–410 (2009). 19759620 10.1038/nature08323 PMC2770190

[b41] Hidaka I., Ohsugi T. & Kubomatsu T. Taste receptor stimulation and feeding behaviour in the puffer, Fugu pardalis I. Effect of single chemicals. Chemical Senses 3, 341–354 (1978).

[b42] Lamb C. F. & Finger T. E. Gustatory control of feeding behavior in goldfish. Physiology & behavior 57, 483–488 (1995). 7753885 10.1016/0031-9384(94)00287-f

[b43] Hara T. Feeding behaviour in some teleosts is triggered by single amino acids primarily through olfaction. Journal of Fish Biology 68, 810–825 (2006).

[b44] Prescott J. *et al.* Hedonic responses to taste solutions: a cross-cultural study of Japanese and Australians. Chemical Senses 17, 801–809 (1992).

[b45] Kolodiy N., Brosvic G. M., Pak D. & Loeffler S. Taste preference behavior in Long-Evans rats and Egyptian spiny mice. Bulletin of the Psychonomic Society 31, 307–310 (1993).

[b46] Caprio J., Shimohara M., Marui T., Harada S. & Kiyohara S. Marine teleost locates live prey through pH sensing. Science 344, 1154–1156 (2014). 24904164 10.1126/science.1252697

[b47] Kasumyan A. O. & DÖving K. B. Taste preferences in fishes. Fish and fisheries 4, 289–347 (2003).

[b48] Blakeslee A. F. Genetics of sensory thresholds: taste for phenyl thio carbamide. Proceedings of the National Academy of Sciences of the United States of America 18, 120 (1932). 16577422 10.1073/pnas.18.1.120 PMC1076171

[b49] Hall J. E. & Guyton A. C. Textbook of Medical Physiology (Elsevier Health Sciences, 2010).

[b50] Horio N. *et al.* Sour taste responses in mice lacking PKD channels. PLoS One 6, e20007 (2011). 21625513 10.1371/journal.pone.0020007 PMC3098277

[b51] Ogawa K. & Caprio J. Citrate ions enhance taste responses to amino acids in the largemouth bass. Journal of neurophysiology 81, 1603–1607 (1999). 10200196 10.1152/jn.1999.81.4.1603

[b52] Ogawa K. & Caprio J. Facial taste responses of the channel catfish to binary mixtures of amino acids. Journal of neurophysiology 82, 564–569 (1999). 10444656 10.1152/jn.1999.82.2.564

[b53] Zhao G. Q. *et al.* The receptors for mammalian sweet and umami taste. Cell 115, 255–266 (2003). 14636554 10.1016/s0092-8674(03)00844-4

[b54] Mueller K. L. *et al.* The receptors and coding logic for bitter taste. Nature 434, 225–229 (2005). 15759003 10.1038/nature03352

[b55] Barretto R. P. J. *et al.* The neural representation of taste quality at the periphery. Nature (2014). 10.1038/nature13873 PMC4297533 25383521

[b56] Kohbara J. & Caprio J. Taste responses to binary mixtures of amino acids in the sea catfish, Arius felis. Chem Senses 21, 45–53 (1996). 8646491 10.1093/chemse/21.1.45

[b57] Ogawa K. & Caprio J. Citrate ions enhance taste responses to amino acids in the largemouth bass. J Neurophysiol 81, 1603–1607 (1999). 10200196 10.1152/jn.1999.81.4.1603

[b58] Ogawa K. & Caprio J. Facial taste responses of the channel catfish to binary mixtures of amino acids. J Neurophysiol 82, 564–569 (1999). 10444656 10.1152/jn.1999.82.2.564

[b59] Ogawa K. & Caprio J. Glossopharyngeal taste responses of the channel catfish to binary mixtures of amino acids. Chem Senses 25, 501–506 (2000). 11015321 10.1093/chemse/25.5.501

[b60] Hyman A. M. & Frank M. E. Effects of binary taste stimuli on the neural activity of the hamster chorda tympani. J Gen Physiol 76, 125–142 (1980). 7411114 10.1085/jgp.76.2.125 PMC2228593

[b61] Maier J. X. & Katz D. B. Neural dynamics in response to binary taste mixtures. J Neurophysiol 109, 2108–2117 (2013). 23365178 10.1152/jn.00917.2012 PMC3628038

[b62] Formaker B. K. & Frank M. E. Responses of the hamster chorda tympani nerve to binary component taste stimuli: evidence for peripheral gustatory mixture interactions. Brain Res 727, 79–90 (1996). 8842385 10.1016/0006-8993(96)00356-3

[b63] Sellier M. J., Reeb P. & Marion-Poll F. Consumption of bitter alkaloids in Drosophila melanogaster in multiple-choice test conditions. Chem Senses 36, 323–334 (2011). 21173029 10.1093/chemse/bjq133

[b64] Niessing J. & Friedrich R. W. Olfactory pattern classification by discrete neuronal network states. Nature 465, 47–52 (2010). 20393466 10.1038/nature08961

[b65] Traxler M. & Gernsbacher M. A. Handbook of psycholinguistics (Academic Press 2011).

[b66] Freedman D. J., Riesenhuber M., Poggio T. & Miller E. K. Categorical representation of visual stimuli in the primate prefrontal cortex. Science 291, 312–316 (2001). 11209083 10.1126/science.291.5502.312

[b67] Akerboom J. *et al.* Optimization of a GCaMP calcium indicator for neural activity imaging. The Journal of neuroscience 32, 13819–13840 (2012). 23035093 10.1523/JNEUROSCI.2601-12.2012 PMC3482105

[b68] Park H.-C. *et al.* Analysis of Upstream Elements in the HuC Promoter Leads to the Establishment of Transgenic Zebrafish with Fluorescent Neurons. Developmental biology 227, 279–293 (2000). 11071755 10.1006/dbio.2000.9898

[b69] Ohki K., Chung S., Ch'ng Y. H., Kara P. & Reid R. C. Functional imaging with cellular resolution reveals precise micro-architecture in visual cortex. Nature 433, 597–603 (2005). 15660108 10.1038/nature03274

