# Supplementary material for: Evolutionary conserved brainstem circuits encode category, concentration and mixtures of taste
Source: Sci Rep. 2015 Dec 7;5:17825. doi: 10.1038/srep17825 (PMC4671064; doi:10.1038/srep17825)
Supplement: Supplementary Information [file srep17825-s1.pdf]

Title: Evolutionary conserved brainstem circuits encode category, concentration and mixtures of taste

Authors: Nuria Vendrell-Llopis[1,2] & Emre Yaksi[1,2,3,4]

Affiliations: 1) NERF, Leuven, Belgium,

2) KU Leuven, Leuven, Belgium

3) VIB, Leuven, Belgium

4) Kavli Institute for Systems Neuroscience and Centre for the Biology of Memory, Norwegian Brain Centre, Norwegian University of Science and Technology (NTNU), Trondheim, Norway.

Contact: emre.yaksi@ntnu.no

Supplementary figure legends

Supplementary figure 1: Reproducibility and robustness of taste responses.

a) An example for the reproducibility/trial-to-trial variability of the taste-evoked neural responses in zebrafish brainstem. **b)** Pair-wise Pearson's correlations representing the trial-to-trial variability between neural responses to different tastants in the same fish as in supplementary figure 1a. We measured reproducibility (solid boxes) by comparing trial-to-trial variability across same tastants. We also measured robustness (dashed boxes) by comparing individual taste trials to the averaged response to 3 trials of same taste. Note that the tastants with prominent responses e.g. sour, bitter compounds and amino-acids have low trial-to-trial variability and high robustness. In all of our analysis we used the 3 trial average of the taste responses to ensure reproducibility. **c)** Left: correlations across trial-to-trial variability for a given tastant, same as solid boxes from supplementary figure 1b, in all fish. Right: correlations of individual trials to the average response of all 3 trials (left), same as dashed boxes from supplementary figure 1b, in all fish. Error bars represent the standard error of the mean..

Supplementary figure 2: Response of brainstem neurons to different tastants

a) Optical section of the facial lobe in zebrafish brainstem expressing GCaMP5 under HuC promoter in most neurons. **b)** Taste-evoked neural responses in the same optical section to all tastes used in this study including the blank control. Response time courses of identified neurons are plotted on the right of each activity map. Neurons responding to at least 1 of the tastants are in black. Neurons that does not respond to any of the tastes are in gray

Supplementary figure 3: Neural representation of taste categories in the brainstem

a) Pair-wise Pearson's correlations between neural responses to different tastants in the brainstem of 10 individual zebrafish. Note that despite the individual variability, our conclusions about the

representation of taste categories are relevant also for the neural data collected/analyzed from individual zebrafish. **b)** Pair-wise Euclidean distances between neural responses to different tastants. Same tastants as in Figure 2.

Supplementary figure 4: Examples for calcium signals of neurons with different dose dependent responses to taste concentrations.

We observed that individual facial lobe neurons can have different types of dose dependent response to quinine-HCl and citric acid. Note that while some example neurons increase their response amplitudes with taste concentration, some other neurons have a non-linear and hard-to-predict relation between their response amplitudes and the increasing taste concentrations. Inset figures correspond to the average dose response curves of neurons resembling the dose response relations of neurons shown by individual examples. We used k-mean clustering to group all responding neurons based on their dose response relations.

Supplementary figure 5: Bitter tastants suppress sour taste responses when mixed together

a) Activity map in response to citric acid, caffeine, denatonium quinine-HCl and citric acid mixed with these bitter substances, all molecules at 5mM each. Relative change in fluorescence intensity is color coded. Please note that quinine-HCl elicit the strongest suppression of citric acid responses when mixed together. White bars are 20 µm. Pair-wise Euclidean distances **(b)** and principle component representations **(c)** of neural responses to different citric acid/quinine-HCl mixtures. Note the sharp transition between the neural representations of taste mixtures both for Euclidean-distances and the first 3 principle component representations, similar to the pair-wise correlation coefficients in Figure 4i.

Supplementary Figure 1

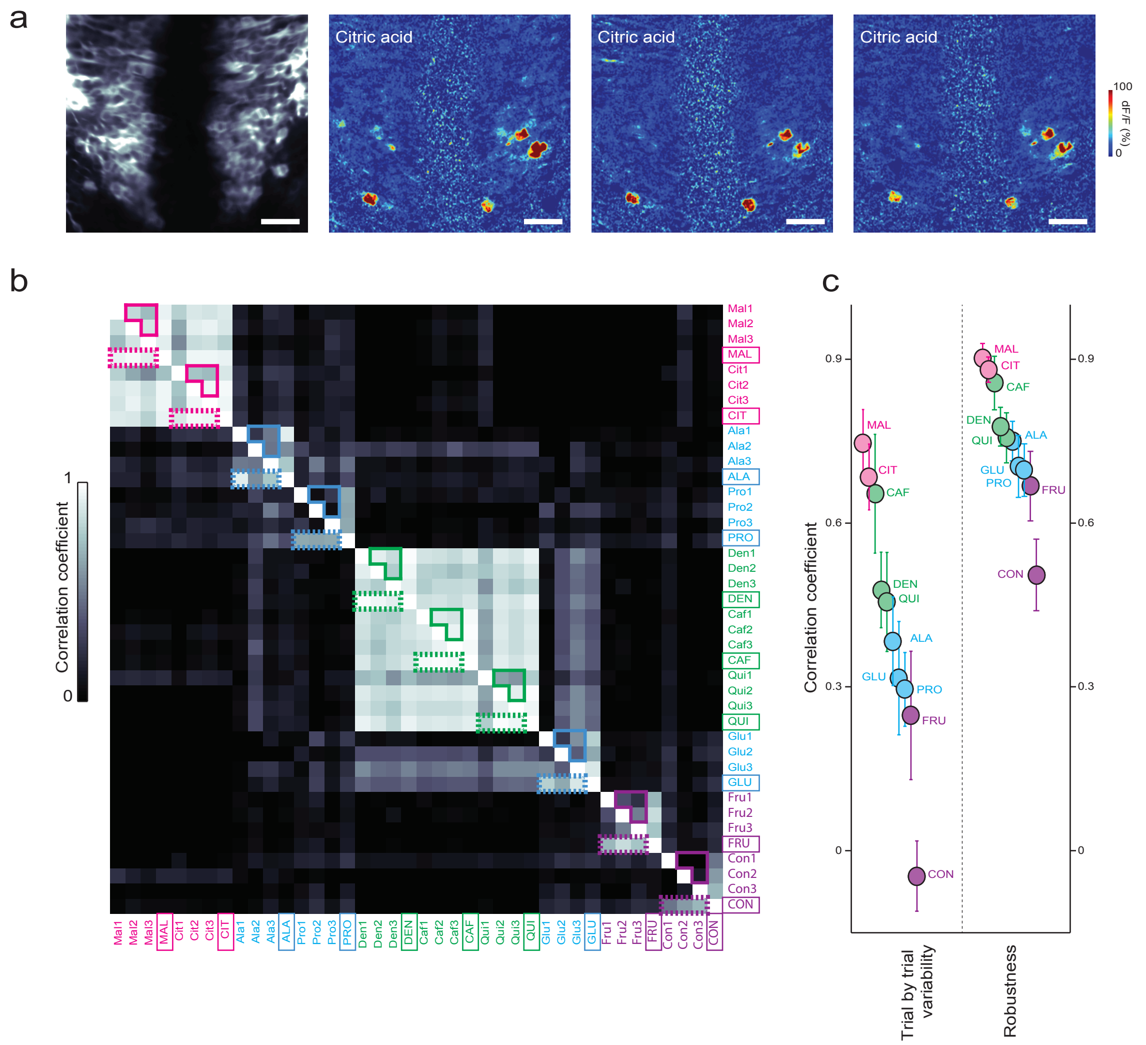

Supplementary figure 1: Reproducibility and robustness of taste responses.
a) An example for the reproducibility/trial-to-trial variability of the taste-evoked neural responses in zebrafish brainstem. **b)** Pair-wise Pearson's correlations representing the trial-to-trial variability between neural responses to different tastants in the same fish as supplementary figure 1a. We measured reproducibility (solid boxes) by comparing trial-to-trial variability across same tastants. We also measured robustness (dashed boxes) by comparing individual taste trials to the averaged response to 3 trials of same taste. Note that the tastants with prominent responses e.g. sour, bitter compounds and amino-acids have low trial-to-trial variability and high robustness. In all of our analysis we used the 3 trial average of the taste responses to ensure reproducibility. **c) Left:** correlations across trial-to-trial variability in individual fish within individual tastants, same as solid boxes from supplementary figure 1b. **Right**: correlations of individual trials to the average response of all 3 trials (left), same as dashed boxes from supplementary figure 1b. Error bars represent the standard error of the mean.

Supplementary Figure 2

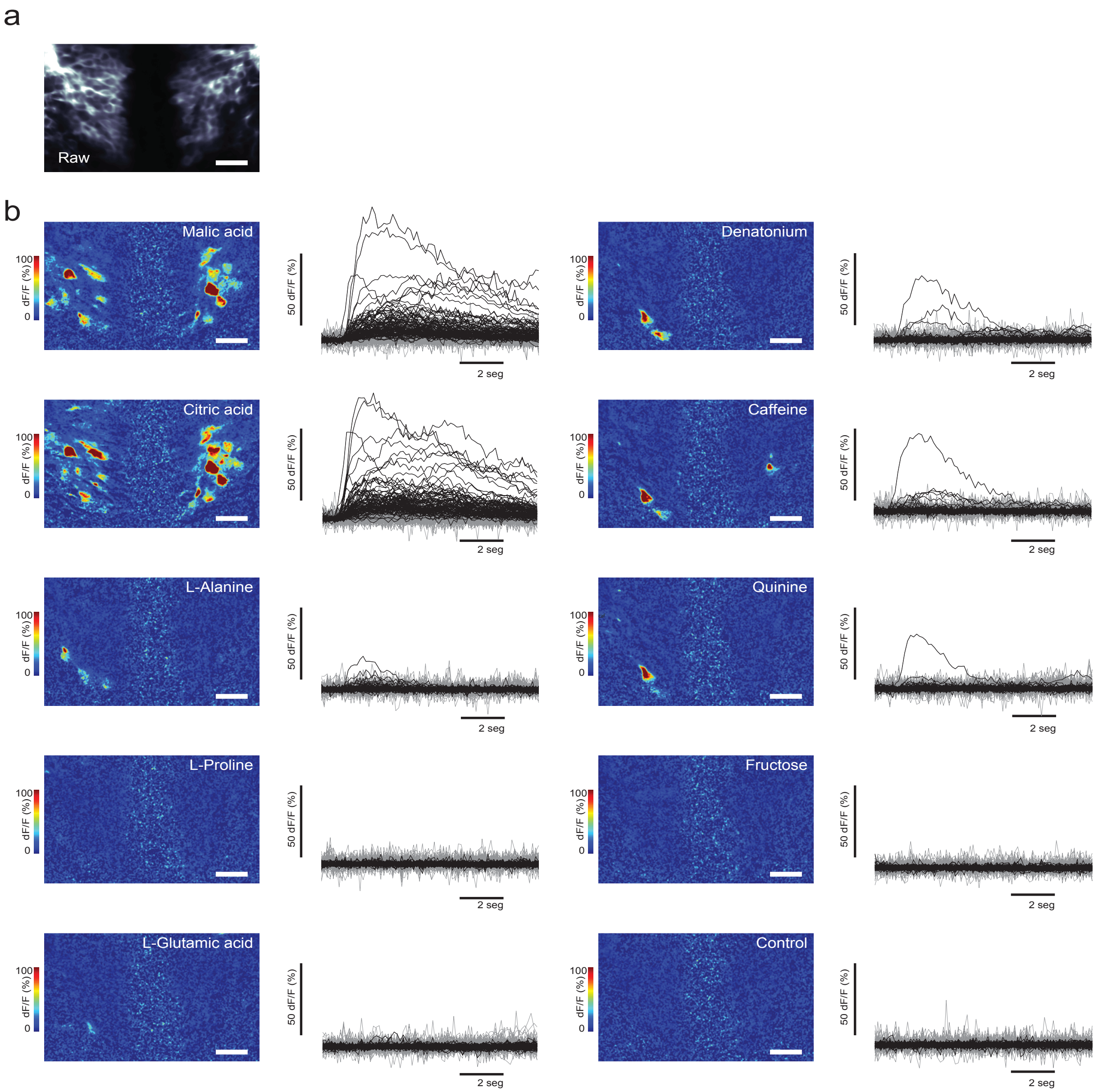

Supplementary figure 2: Response of brainstem neurons to different tastants
a) Optical section of the facial lobe in zebrafish brainstem expressing GCaMP5 under HuC promoter in most neurons.
b) Taste-evoked neural responses in the same optical section to all tastes used in this study including the blank control. Response time courses of identified neurons are plotted on the right of each activity map. Responding neurons are in black, neurons classified as non-responsive are in gray.

Supplementary figure 3

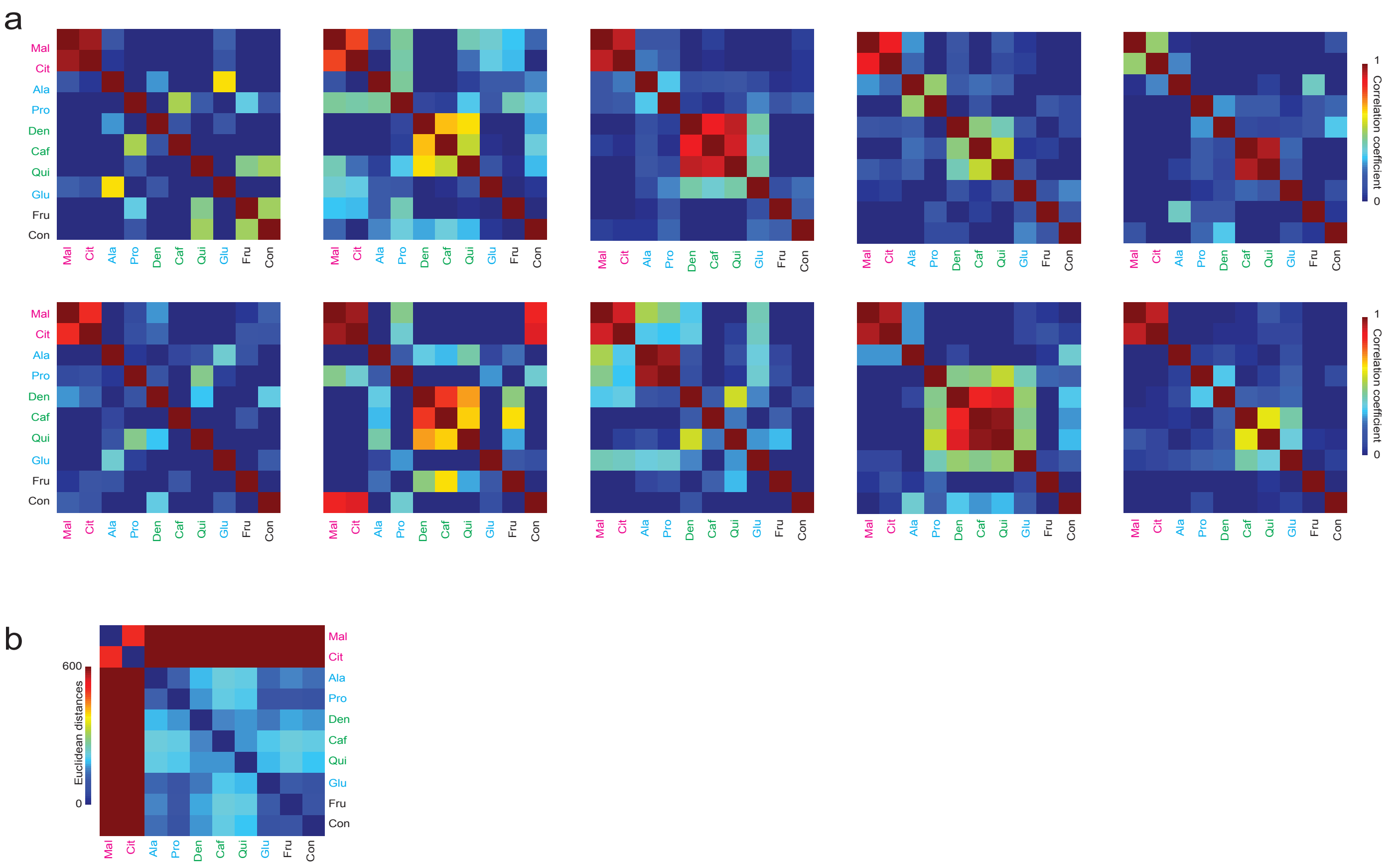

Supplementary figure 3: Neural representation of taste categories in the brainstem
a) Pair-wise Pearson's correlations between neural responses to different tastants in the brainstem of 10 individual zebrafish. Note that despite the individual variability, our conclusions about the representation of taste categories are relevant also for the neural data collected/analyzed from individual zebrafish. **b)** Pair-wise Euclidean distances between neural responses to different tastants. Same tastants as in Figure 2.

Supplementary figure 4

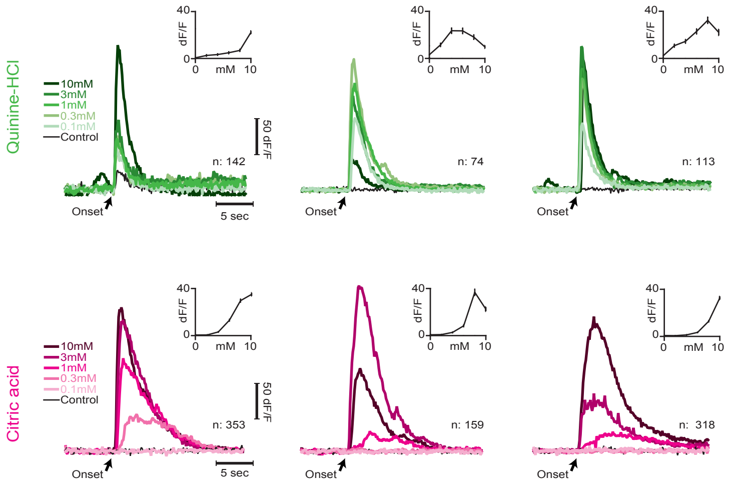

Supplementary figure 4: Examples for calcium signals of neurons with different dose dependent responses to taste concentrations.
We observed that individual facial lobe neurons can have different types of dose dependent response to quinine-HCl and citric acid. Note that while some example neurons increase their response amplitudes with taste concentration, some other neurons have a non-linear and hard-to-predict relation between their response amplitudes and the increasing taste concentrations. Inset figures correspond to the average does response curves of neurons resembling the dose response relations of neurons shown by individual examples. We used k-mean clustering to group all responding neurons based on their dose response relations.

Supplementary figure 5

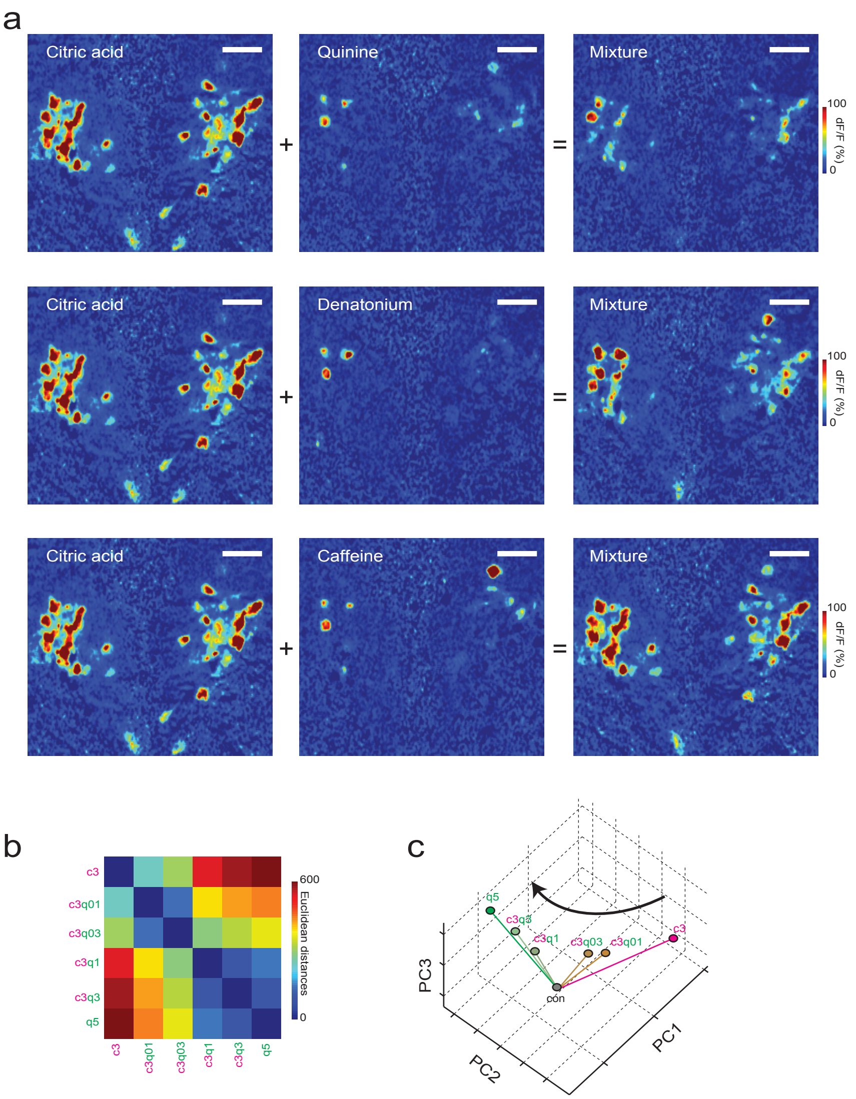

Supplementary figure 5: Bitter tastants suppress sour taste responses when mixed together
a) Activity map in response to citric acid, caffeine, denatonium, quinine-HCl and citric acid mixed with these bitter substances, all molecules at 10mM each. Relative change in fluorescence intensity is color coded. Please note that quinine-HCl elicit the strongest suppression of citric acid responses when mixed together. White bars are 20 μm. Pair-wise Euclidean distances **(b)** and principle component representations **(c)** of neural responses to different citric acid/quinine-HCl mixtures. Note the sharp transition between the neural representations of taste mixtures both for Euclidean-distances and the first 3 principle component representations, similar to the pair-wise correlation coefficients in Figure 4i

Supplementary Table 1

Categories

Tastant	Ph
Malic acid 10mM	2.78
Citric Acid 10mM	2.62
L-Alanine 100mM	5.61
L-Proline 100mM	5.48
Caffeine 10mM	5.64
Denatonium 10mM	6.16
Quinine-HCl 10mM	6.14
L-Glutamic acid 100mM	6.59
Fructose 100mM	5.64

Concentrations

Tastant	Ph
Citric acid 10mM	2.65
Citric acid 3mM	2.97
Citric acid 1mM	3.19
Citric acid 0.3mM	3.58
Citric acid 0.1mM	4.01

Tastant	Ph
Quinine-HCl 10mM	6.02
Quinine-HCl 3mM	6.14
Quinine-HCl 1mM	6.03
Quinine-HCl 0.3mM	5.94
Quinine-HCl 0.1mM	6.01

Mixtures

Tastant	Ph
Citric Acid 5mM	2.75
Citric Acid 1mM	3.16
Citric Acid 0.5mM	3.22
Malic Acid 5mM	2.91
Malic Acid 1mM	3.38
Malic Acid 0.5mM	3.53
Citric Acid 5mM + Malic Acid 5mM	2.83
Citric Acid 1mM + Malic Acid 1mM	3.09
Citric Acid 0.5mM + Malic Acid 0. 5mM	3.24

Tastant	Ph
Citric Acid 5mM	2.74
Citric Acid 1mM	3.20
Citric Acid 0.5mM	3.24
Quinine-HCl 5mM	6.09
Quinine-HCl 1mM	6.02
Quinine-HCl 0.5mM	6.11
Citric Acid 5mM + Quinine-HCl 5mM	3.30
Citric Acid 1mM + Quinine-HCl 1mM	3.41
Citric Acid 0.5mM + Quinine-HCl 0. 5mM	3.58

Tastant	Ph
Citric acid 3mM	2.89
Citric acid 3mM + Quinine-HCl 0.1mM	2.85
Citric acid 3mM + Quinine-HCl 0.3mM	2.89
Citric acid 3mM + Quinine-HCl 1mM	3.08
Citric acid 3mM + Quinine-HCl 3mM	3.49
Quinine-HCl 5mM	6.07

Supplementary Table 2

Figure	Test	Sample/experiment			p value
2i	Mann-Whitney U-test.	12 fish, each taste repeated 3 times	TBF	amino acid/bitter	1.41E-04
				sour/bitter	7.02E-07
				control/bitter	4.47E-07
				amino acid/sour	8.55E-10
				control/sour	1.18E-06
				control/amino acid	0.00903
			ATS	amino acid/bitter	5.12E-04
				sour/bitter	2.58E-08
				control/bitter	6.42E-06
				amino acid/sour	2.71E-10
				control/sour	1.13E-06
				control/amino acid	0.0162
3e	wilcoxon paired test (signrank)	15 fish, each taste repeated 3 times	ATS-Citric acid	5mM - 1mM	6.10E-05
				5mM - 0.5mM	6.10E-05
				1mM - 0.5mM	0.0413
				5mM - control	6.10E-05
				1mM - control	6.10E-05
				0.5mM - control	6.10E-05
			ATS-Quinine	5mM - 1mM	0.002
				5mM - 0.5mM	0.0026
				1mM - 0.5mM	0.7615
				5mM - control	6.10E-05
				1mM - control	0.0151
				0.5mM - control	0.0107
4c-left	wilcoxon paired test (signrank)	7 fish, each taste repeated 3 times	SUPPRESSION	0.5mM 1mM	0.6791
				0.5mM-5mM	0.0347
				5mM - 1mM	0.0778
			SYNERGY	0.5mM 1mM	0.0526
				0.5mM-5mM	0.001
				5mM - 1mM	0.004
4c-right	wilcoxon paired test (signrank)	7 fish, each taste repeated 3 times	SUPPRESSION	0.5mM 1mM	0.0295
				0.5mM-5mM	8.63E-04
				5mM - 1mM	0.0486
			SYN	0.5mM 1mM	0.0099
				0.5mM-5mM	5.02E-04
				5mM - 1mM	2.20E-03
4e-left	wilcoxon paired test (signrank)	7 fish, each taste repeated 3 times	SUP	0.5mM 1mM	0.2461
				0.5mM-5mM	0.0017
				5mM - 1mM	0.0127
			SYN	0.5mM 1mM	0.2762
				0.5mM-5mM	0.002
				5mM - 1mM	0.02
4f	wilcoxon paired test (signrank)	15 fish, each taste repeated 3 times	TBF	mixture1/citric acid1	0.3028
				mixture 1/quinine1	0.0302
				mixture 1/control	1.22E-04
			ATS	mixture 1/ citric acid 1	8.55E-04
				mixture 1/quinine1	0.00403
				mixture 1/control	6.10E-05
